# Infection of *Leishmania donovani* in *Phlebotomus orientalis* Sand Flies at Different Microhabitats of a Kala-Azar Endemic Village in Eastern Sudan

**DOI:** 10.3390/tropicalmed9020040

**Published:** 2024-02-02

**Authors:** Altayeb Khogali, Dia-Eldin A. Elnaiem, Ramón Díaz-Regañón, Tayseer Jibreel, Bakri Y. M. Nour, Samira Hamid Abdelrahman, Ricardo Molina, Maribel Jiménez

**Affiliations:** 1 Blue Nile National Institute for Communicable Diseases, University of Gezira, Wad Medani 21111, Sudan; altaiebali87@gmail.com (A.K.); taiseer166@gmail.com (T.J.); bakrinour@gmail.com (B.Y.M.N.); samhamid2002@yahoo.co.uk (S.H.A.); 2 Department of Natural Sciences, University of Maryland Eastern Shore, 1 Backbone Rd., Princess Anne, MD 21853, USA; 3 Medical Entomology Laboratory, National Center for Microbiology, Instituto de Salud Carlos III, 28220 Majadahonda, Madrid, Spain; r.diaz@isciii.es (R.D.-R.); rmolina@isciii.es (R.M.); 4 Collaborative Biomedical Research Center in Infectious Diseases (CIBERINFEC), Instituto de Salud Carlos III, 28029 Madrid, Spain

**Keywords:** visceral leishmaniasis, *Leishmania donovani*, infection rates, *Phlebotomus orientalis*, sand flies, Sudan, East Africa

## Abstract

A study was carried out to compare the infection rates of *Leishmania donovani* in *Phlebotomus orientalis* sandflies at different microhabitats of a VL endemic village in Gedarif state, Sudan. DNA extracts of 1078 *P. orientalis* sand fly females sampled by CDC light traps from indoor, outdoor, peri-domestic, and sylvatic sites, in three transmission seasons, March–June 2016–18, in Helat-Belo village, were subjected to independent PCR amplifications targeting *Leishmania* kDNA and the *cpb* gene followed by ITS1 region sequencing. *Leishmania* kDNA was detected in 1.4% of the 1078 *P. orientalis* females captured in the area. Two of these specimens showed a characteristic 741 bp band of *L. donovani* after *cpb* gene amplification. The DNA sequence of the ITS1 region of the parasites matched the ITS1 *L. donovani* genotype F. There were no signficant differences between rates of infection of *L. donovani* in *P. orientalis* captured at different sites. Blood meals found in infected flies origninated from human (5 specimens), cattle (4 specimens) and donkey (2 specimens). The finding of fresh cow and donkey blood in the infected flies suggests the possible role of these animals in the zoopotentiation and/or zooprophylaxis against VL. The study provides important information for VL transmission models and control programs in East Africa.

## 1. Introduction

Leishmaniases are poverty-related vector-borne neglected tropical diseases caused by infection with *Leishmania* (Kinetoplastida: Trypanosomatidae) parasites, which are transmitted between mammalian hosts by female phlebotomine sand flies (Diptera: Psychodidae) [1]. More than 20 species of *Leishmania* infect humans, resulting in cutaneous, mucocutaneous, and visceral leishmaniasis [2].

Visceral leishmaniasis (VL), also known as kala-azar, is the most serious form of leishmaniasis. Caused by *Leishmania donovani* in Asia and East Africa and by *Leishmania infantum* in Latin America and the Mediterranean area, VL is usually fatal if untreated [1]. The disease results from systemic infection by *Leishmania* amastigotes in the liver, spleen, bone marrow, and the lymphatic system [3]. Symptoms of VL include irregular fever, anemia or pancytopenia, marked weight loss, ataxia, and splenomegaly at late stages [4].

Currently, there is an estimated annual incidence of 50,000 to 90,000 new cases of VL in the world, with only 25–45% that are reported to the WHO [5]. Previous literature showed that about 90% of VL cases occur in just six countries: India, Bangladesh, Sudan, South Sudan, Brazil, and Ethiopia, with the highest disease burden in Southeast Asia [3]. However, due to successful control of VL in the Indian subcontinent, East Africa currently carries the highest burden of the disease, accounting for 66% of worldwide incidence [6]. Within this region, the most affected countries are Sudan, Republic of South Sudan, and Ethiopia [1].

Transmission of *L. donovani* in East Africa is maintained by three sand fly species, *Phlebotomus orientalis* in northern foci in Ethiopia, Republic of South Sudan (RSS), and Sudan and *Phlebotomus martini* and *Phlebotomus celiae* in southern foci in Ethiopia, Kenya, and RSS [7,8,9]. *Phlebotomus orientalis* is the predominant vector of VL in East Africa [9,10,11]. Studies showed that this sand fly species has relatively high infection rates of *L. donovani* in several sites in Sudan [12,13,14,15]. Microhabitat distribution studies showed that the vector is highly associated with black cotton soil (vertisol) and *Acacia-seyal* and/or *Balanites aegyptiaca* trees [9,16,17]. In VL-endemic villages and their surrounding microhabitats, *P. orientalis* is more abundant in sylvatic and peri-domestic sites than in outdoor and indoor sites [10,18]. Several studies in Ethiopia demonstrated that this vector preferentially feeds on cows and donkeys rather than humans and other animals [19]. Using host choice experiments and molecular identification of blood meals, we found similar results for *P. orientalis* in villages of eastern Sudan [20].

Although vector bionomic studies have provided clear information about the distribution and the host preference of *P. orientalis* sand flies [7,8,9,18,19,20,21,22,23,24]. Little is known about the rates of infection of *L. donovani* at village microhabitats. This missing information is of special importance for constructing transmission models and designing control measures to reduce the risk of acquiring the disease. In this study, we compare the infection rates of *L. donovani* in *P. orientalis* captured at sylvatic, peri-domestic, outdoor and indoor microhabitats in a kala-azar-endemic village in eastern Sudan.

## 2. Materials and Methods

### 2.1. Study Area

**Figure 1:**
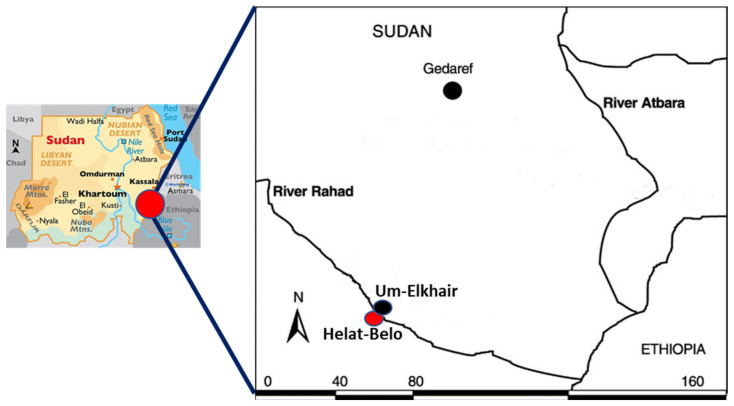
Map of Gedarif state (Sudan), showing the location of Helat-Belo village (Nour-Elmadina).

Sand flies were collected with CDC light traps from March to June in 2016, 2017, and 2018 in Helat-Belo (Belo), also known as Nour-Elmadina (12°52′476″ N 035°09′039″ E), which is located on the southern bank of the River Rahad, ~120 km southwest of Gedarif town (Figure 1). This village, which has a total of 1700 inhabitants, was selected for the study because of its consistently high incidence rate of VL and its location at the border of Dinder National Park, an inhabited game reserve known for sylvatic transmission of the disease [14,25]. According to records of the local kala-azar treatment center in Um-Elkhair (Gedarif state Ministry of Health), the total number of VL cases reported from Belo village in 2016, 2017, and 2018 were 82, 120, and 73, respectively.

Belo village is surrounded by Dinder National Park (DNP) woodland on the east, south, and southwest and the seasonally flowing Rahad river from the east, west, and north. The ecology of the village has been described in previous publications [11,18,20,26]. The topography is a flat plain covered by black cotton soil (vertisols). Other soils, which occupy small fractions of the area, include a mixture of alluvial clays, silts, and sands of varying depths on the banks of the Rahad river. The climate of the region is tropical continental, with an estimated annual rainfall of 400–1400 mm. The year is sharply divided between the rainy season, June–October, and the dry season, November–May. According to readings at Samsam Meteorological station, located 15 km N of Belo village, the daily mean minimum temperature is 26.5 °C in the rainy season and 25.7 °C in the dry season; corresponding maxima are 36.4 °C and 39.1 °C, respectively (https://weatherandclimate.com; accessed on 16 January 2024). The natural vegetation of the area is dry savanna woodland. The main indigenous trees in the region are *Balanites aegyptiaca* (known locally as “hig-leeg”), *Acacia seyal* (“Taleh”), *Acacia senegal* (“Hashab”), *Acacia mellifera* (“Kiter”), *Combretum* spp., *Calotropis procera* (“Usher”), as well as some riverine vegetation consisting of *Hyphaena thaibaica*, *Zyzyphus spina-christa*, and other trees and bushes. Along the Rahad riverbanks, some fruit orchards are found. Dura (*Sorghum pupura*), sesame (*Sesamum orientate*), Dokhon (*Pennisetum typhodium*), and groundnuts (*Arachis hypogaea*) are grown as cash crops over extensive areas.

The human population of Belo village belongs to the Fulani group, who have a recent history of settlement in the region.

**Figure 2:**
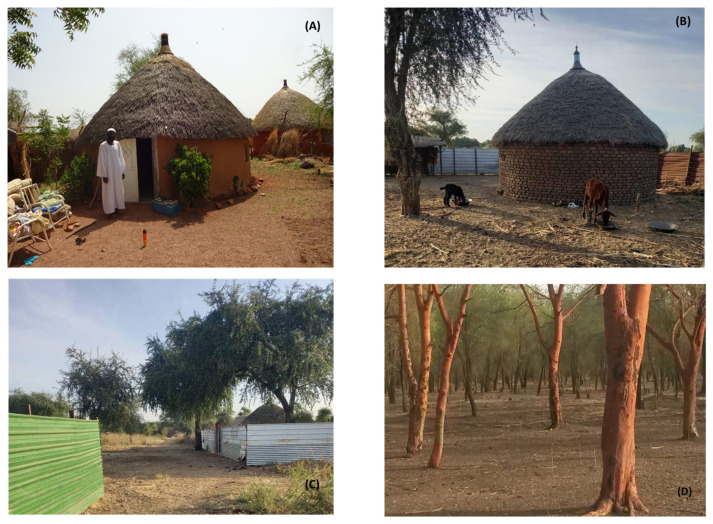
Microhabitats at sand fly collection sites in Helat-Belo villages, Gedarif state, eastern Sudan. (**A**) Indoor sites; (**B**) Outdoor sites; (**C**) Peri-domestic habitats, separated from outdoor habitats by 1.5 m high metal fences; (**D**) Sylvatic sites showing *Acacia seyal* trees.

In Gedarif state, the village microhabitats consist of 4 distinct types that can be of special significance to the epidemiology and control of VL [11]. The first microhabitat, referred to as indoor, is the inside of the rooms which are African huts, constructed of mud/brick or thatched-grass walls (Figure 2A). These rooms are used by people to keep their belongings and spend the nights during the rainy or cold nights in autumn (July–October) or winter (November–February). The second microhabitat, denoted as outdoor, is the “courtyard” area of the house, which is separated from the peri-domestic area by 1.5–2.00 m reed or metal fences, built to provide privacy (Figure 2B). People usually spend the nights in this outdoor area during the hot summer months (March–June), which coincide with the man-biting activity of *P. orientalis* and therefore the transmission season of VL. During the nighttime, people protect their domestic and livestock animals by keeping them in this fenced outdoor area. The third microhabitat, denoted as peri-domestic, lies in the immediate surroundings of the houses, outside the fenced area, and consists of roads and open spaces within the village (Figure 2C). The fourth microhabitat, labelled as sylvatic, lies outside the village and is characterized by dense thickets of trees, mainly *A. seyal* and *B. aegyptiaca* (Figure 2D).

### 2.2. Sand Fly Collection Sites and Protocol

**Figure 3:**
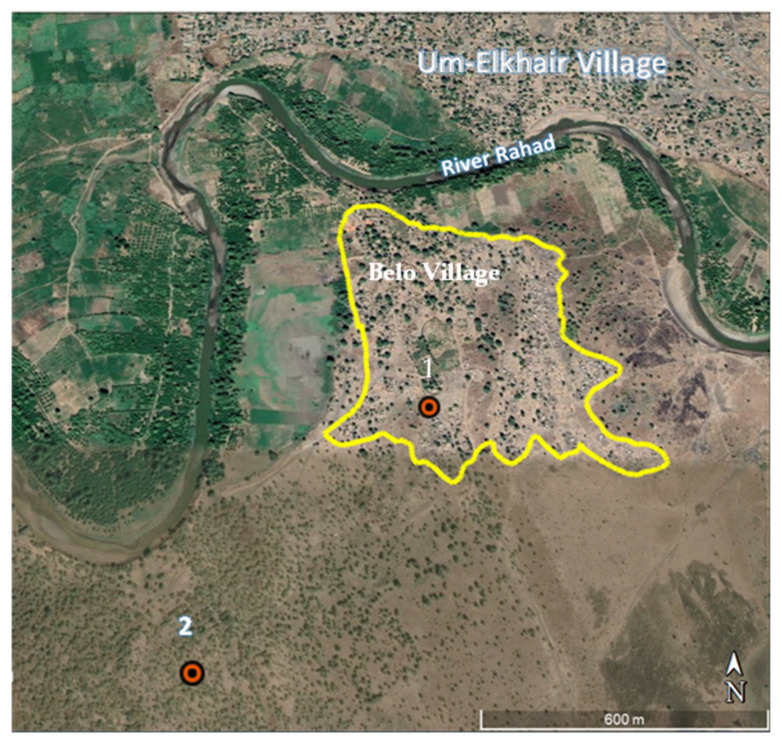
Location of sites used for collection of sand flies at Belo-village area, Gedarif state, Sudan. 1 = indoor, outdoor, peri-domestic sites; 2 = sylvatic sites. Polygon shows the boundary of the village. The map was created using Google Earth Pro Version 7.3.

Collections of sand flies were conducted simultaneously at the sylvatic, peri-domestic, outdoor, and indoor sites (Figure 2 and Figure 3) of the study area for 4–8 nights/month during the peak *P. orientalis* sand fly season (March–June; [9,18]) in 2016, 2017, and 2018. The total numbers of trapping nights in March, April, May and June were 36, 26, 36, and 30, respectively. The indoor, outdoor, and peri-domestic collection sites were based on two adjacent houses with the following description. House 1 consisted of two brick huts surrounded with metal fence on two sides, reed fence on a third side, while the fourth side was left completely open to the peri-domestic area. House 2 also consisted of two brick huts, but it was surrounded by metal fence on all sides. The metal fences of both houses were raised at one foot above ground level to prevent corrosion. The sylvatic site was located in a thicket of *A. seyal*, about 600 m from the southern edge of the village (Figure 3). This thicket of trees is a natural extension of the dense woodland of Dinder National Park, which lies 5 km south of the village.

Sand fly sampling was made from inside one hut from each house (2 indoor sites), courtyards of the two houses (2 outdoor sites), the immediate peri-domestic area near the walls of each of the two houses (2 peri-domestic sites), and two sites in the sylvatic microhabitat. The indoor, outdoor, and peri-domestic sites were separated by 50 m from each other and by 800 m from the two sylvatic sites (Figure 3). In each microhabitat, sand flies were collected using 2 CDC traps that were set between 18:00–06:00 HR, at 0.5 m above the ground level.

### 2.3. Sand Fly Collection, Identification and DNA Extraction

Sand flies that were captured in the CDC traps were killed by aspiration into 14 mL falcon tubes containing 70% ethanol. These specimens and other sand fly females found dead in the traps were preserved in freshly prepared 70% ethanol at ambient temperature. The gonotrophic status of sand flies selected for screening for *Leishmania* infection varied in different years. In 2016 and 2017, only blood-fed and gravid females were screened for *Leishmania* infection. In 2018, random samples of unfed and blood-fed flies were screened for *Leishmania* infection.

In the laboratory, sand flies preserved in 70% ethanol were washed individually in sterile distilled water and placed in ELISA micro titer plates. Afterwards, the head, wings, genitalia, and legs of each female sand fly were removed. The genitalia and the head were processed for taxonomical identification, following standard taxonomic keys and protocols [27,28]. The presence of a blood meal was determined by observation under a stereomicroscope. The thorax and abdomen of each sand fly were used for DNA extraction using a Speedtools Tissue DNA Extraction Kit (Biotools^®^ B &M Labs S.A., Madrid, Spain) according to the manufacturer’s instructions. DNA samples were stored at −20 °C until use for PCR.

### 2.4. Molecular Detection of Leishmania spp.

The DNA extracts of *P. orientalis* were subjected to PCR amplification targeting the conserved 120 bp region of *Leishmania* kinetoplast DNA (kDNA). The primers used in this amplification were JW11 (forward) 5′-CCT ATT TTA CAC CAA CCC CCA GT-3′ and JW12 (reverse) 5′-GGG TAG GGG GGT TCT GCG AAA-3′ [29] and conditions following the protocols previously described [28]. Each PCR reaction consisted of 2 µL of 1× buffer, 0.8 µL of MgCl_2_ (2 mM), 0.2 µL of 100 U/mL dNTPs, and 0.5 µL of HotSplit polymerase (final concentration 2.5 U/mL), all reagents being from Biotools (Biotools^®^ B&M Labs S.A., Madrid, Spain), 1 µL of JW11 and 1 µL of JW12 (final concentration 0.5 pmol each), 0.8 µL of BSA (final concentration 0.8 mg/mL, 20 mg/mL, Roche^®^, Basel, Switzerland), and a variable volume of DNA adjusted to 30 ng according to the concentration values of the sample obtained by the NanoDrop™, Wilmington, DE, USA. DNase-free water was then added to make up a final volume of 20 µL. Positive controls were 1 µL of DNA (10 pg DNA/µL) of *L. donovani*.

The PCR conditions used were: initial denaturation at 94 °C for 6 min, followed by 40 cycles of denaturation at 94 °C for 30 s, annealing at 53 °C for 45 s, and elongation at 72 °C for 1 min. Finally, the final elongation was carried out at 72 °C for 10 min.

All PCR products were subjected to electrophoresis on 1.5% agarose gel (Conda^®^, Torrejón de Ardoz, Madrid, Spain) stained with 5 µL of PronaSafe (Conda^®^, Torrejón de Ardoz, Madrid, Spain), and visualized under UV light, and photographed.

### 2.5. Amplification of the cpb Gene

Original DNA samples that were positive by kDNA-PCR were subjected to a confirmatory PCR amplification targeting the 741 bp and 702 bp of the *cpb* gene which are specific for *L. donovani* and *L. infantum,* respectively [30]. The amplification was performed as described before [31,32]. The master mix was prepared in a final volume of 50 μL containing: 5 μL of 10× PCR buffer for HotSplit polymerase, 0.5 μL HotSplit polymerase (5U/μL), 1 μL of dNTPs 10 mM, 0.2 mM of each deoxynucleoside triphosphate, and 3 μL of 50 mM MgCl_2_ (Biotools^®^ B&M Labs S.A., Madrid, Spain), 2 μL of BSA DNAse Free (20 mg/mL, Roche^®^, Basel, Switzerland), and 40 pmol of each primer cpbE 5′-CGT GAC GCC GGT GAA GAA T-3′ y cpbF 5′-CGT GCA CTC GGC CGT CTT-3′. Sixty nanograms of DNA was used in each reaction. The PCR reaction conditions were 1 cycle at 96 °C for 6 min, then 40 cycles (94 °C for 30 s, 62 °C for 1 min, 72 °C for 1 min), followed by 1 cycle at 72 °C for 10 min. PCR products were analyzed by electrophoresis in 1.5% agarose gels, stained with 5 µL of PronaSafe (CONDA^®^, Torrejón de Ardoz, Madrid, Spain), and visualized by UV illumination.

### 2.6. Characterization of Leishmania Species

To characterize the *Leishmania* species of each of the infected *P. orientalis*, the ITS1 (internal transcribed spacer 1) of ribosomal RNA were amplified by PCR. The primers MEST1 (forward) 5′-CTG GAT CAT TTT CCG ATG-3′ and MEST2 (forward) 5′-TGA TAC CAC TTA TCG CAC TT-3′ were used [33]. Each reaction contained 5 µL of 1× buffer, 1.5 µL of MgCl_2_ (1.5 mM), 1 µL of 200 mM dNTPs, 1 µL of MEST1 (final concentration 25 pmol), 1 µL of MEST2 (final concentration 25 pmol), 2 µL BSA (final concentration 0.8 mg/mL), 0.5 µL HotSplit polymerase (final concentration 2.5 U/µL), and 60 ng of DNA- and DNAase-free water to complete a reaction volume of 50 µL. A negative control was included in each PCR preparation to confirm the absence of contaminating DNA, and a positive control with 1 µL of DNA (1 ng DNA/µL) of *L. donovani* was used. The PCR conditions used were an initial denaturation at 94 °C for 6 min, followed by 40 cycles of denaturation at 95 °C for 20 s, annealing at 53 °C for 30 s, and elongation at 72 °C for 1 min. Final elongation was done at 72 °C for 10 min. PCR products were separated on 1.5% agarose gel (Conda^®^, Madrid, Spain) and stained with “Pronasafe Nucleic Acid Staining Solution” (10 mg/mL) (Conda^®^, Madrid, Spain). Then, the bands were visualized under UV light and excised from the gel under UV exposure.

The excised PCR product was directly purified using the SpeedTools PCR Clean-up kit (Biotools^®^ B&M Labs, Madrid, Spain) and then sequenced in an ABI PRISM 3730XL DNA Analyzer (Applied Biosystems^®^, Foster City, CA, USA, EEUU). The electropherograms were manually inspected and corrected using ChromasPro program (McCarthy, Queensland, Australia). Nucleotide sequences obtained were analyzed with the DNASTAR program (Lasergene v7.1^®^, Madison, WI, USA). Nucleotide sequences data reported in this paper were deposited at the DDBJ database (http://www.ddbj.nig.ac.jp; accessed on 20 December 2023).

To prevent PCR contamination, sample preparation, reaction set-ups, and PCR amplifications were performed in separate rooms, using different lab coats and gloves.

### 2.7. Blood Meal Identification

Blood meal identification of engorged sand flies was conducted by the amplification of a fragment of 359 bp of the vertebrate cytochrome b (*cyt* b) gene with universal degenerated primers, followed by sequencing as described before [32]. The detailed data of *P. orientalis* host preferences at different microhabitats were published previously [20].

### 2.8. Statistical Analysis

Comparisons of infection rates of *L. donovani* in *P. orientalis* sand flies captured at different microhabitats were subjected to Chi-square and Fisher Exact tests within SPSS Version 28 (IBM^®^). Bonferroni correction was applied to the post hoc pairwise multiple comparisons. The Fisher Exact test was also used to compare the overall infection rate inside and outside the village.

## 3. Results

A total of 1078 female *P. orientalis* (754 unfed and 324 blood-fed/gravid females), collected from different sites of the Helat- Belo area, were subjected to kDNA-PCR, followed by *cpb* gene amplifcation to detect *Leishmania* infections and species in the vector and then characterize the genotype of the parasite. The number of sand flies, their gonotrophic status, and rates of infection with the parasite are shown in Table 1. *Leishmania* kDNA was detected in 15 out of the 1078 (1.4%) *P. orientalis* females captured at the different microhabitats of the village. In a descending order, the highest infection rates were found in sand flies captured in the house yards (outdoor sites, 11/439 = 2.5%), followed by sylvatic sites (3/236 = 1.7%) and then the indoor site (1/202 = 0.5%). None of the 201 sand flies captured at the peri-domestic sites was found infected with *Leishmania* parasites.

The statistical analysis showed an overall significant association between the microhabitat type and the infection rates of *L. donovani* in *P. orientalis* (Pearson Chi-square = 8.016, df = 3, *p* = 0.046). However, post hoc pairwise Fisher Exact tests, adjusted with Bonferroni correction for multiple comparisons, showed no signficant differences in infection rates at different microhabitats (Table 2). Similarly, there were no significant differences between the infection rates of *L. donovani* in *P. orientalis* females captured outside the village (sylvatic site) and inside the village (total of all flies at indoor, outdoor, and peri-domestic sites).

**Figure 4:**
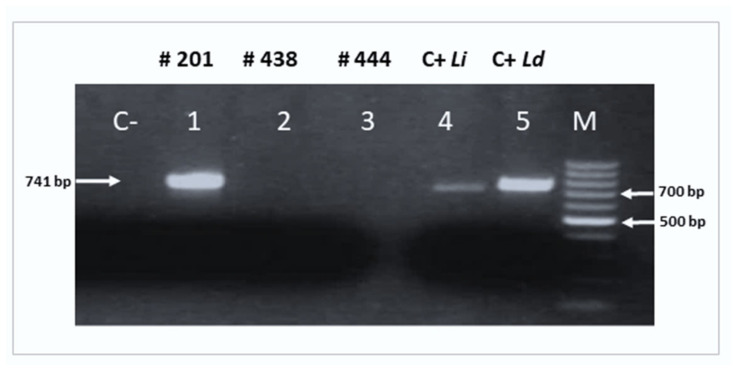
PCR products of the *cpb* gene of *Leishmania* parasites detected in a *P. orientalis* female captured at an outdoor site in Belo village (Gedarif state, Sudan). A specific band of 741 bp corresponding to *L. donovani* was observed in lane 1 (# 201). Lane 2 (# 438) and lane 3 (# 444), both of them previously positive to kDNA, were negative to the *cpb* gene. Lane 4 and Lane 5 were positive controls containing 1 µL of 10 pg DNA/µL PCR products of *L. infantum (Li*, 702 bp) and *L. donovani* (*Ld*, 741 bp). Lane M: 100 bp molecular weight marker (Biotools^®^).

Amplifications of the *cpb* gene and ribosomal internal transcribed spacer ITS1 region were done in four (#201, #438, #444, and #305) out of the 15 samples that were positive to kDNA PCR. Following *cpb* gene amplification, one of these infected sand flies (#201) showed a characteristic 741 bp band of *L. donovani* (Figure 4). On the other hand, amplification of the ITS1 region was successful in two samples (#201 and #305; DDBJ database (http://www.ddbj.nig.ac.jp; accessed on 20 December 2023); accession no LC782821 and LC791472, respectively), both of them matching the *L. donovani* complex. Interestingly, the ITS1 sequence of #201 matched *L. donovani* genotype F presenting four polymorphic microsatellites (2C, 9A, 5TA, and 7A) found in *L. donovani* from Sudan [34].

Amplification and sequencing of the cytochrome b gene fragment in the engorged sand flies showed that five, four, and two of the infected *P. orientalis* females were fed on human (*Homo sapiens*), cow (*Bos taurus*), and donkey (*Equus asinus*) blood, respectively. The other unfed and gravid infected females had no detectable blood meal.

## 4. Discussion

VL continues to cause a major threat to the health and welfare of marginalized populations in East Africa. Some of the highest endemic East African VL foci are found in Gedarif state, eastern Sudan, where thousands of cases of the disease occur annually among relatively small populations residing near the Rahad and Atbara seasonal rivers [26,35]. As in other vector-borne diseases, control of this disease relies on a clear understanding of its epidemiology and delineation of places and times where and when transmission of the parasite takes place and where people contract the infection. Furthermore, accurate information on rates of infection of *L. donovani* in the vector are crucial for building mathematical models to understand the transmission dynamics, predict the numbers of cases, and planning control programs.

A number of authors have shown variable rates of infection of *L. donovani* in wild-caught phlebotomine sand flies in East Africa. Hoogstraal et al. [12] and Hoogstraal & Dietlein [13] reported infection rates ranging between 1.9% and 5% and averaging 2.5% in 4553 *P. orientalis* flies captured in 1962–1964 in woodland of the Paloich area of the Republic of South Sudan [7,36]. Similarly, high infection rates of *L. donovani*, ranging from 3.4% [25] to 6.9% [14], were found in *P. orientalis* captured from uninhabited woodland in Dinder National Park, eastern Sudan, during the dry seasons of 1995–1996 and 1998–1999, respectively. Hassan et al. [37] encountered a similarly high infection rate (8.6%) of *L. donovani* in *P. orientalis* in the dry season of 2005, in the same sylvatic sites in Dinder National Park. Using microscopy and molecular typing, Elnaiem & Osman [38] obtained the first evidence for active transmission of *L. donovani* in human habitations in eastern Sudan by finding the parasite in one out of 12 *P. orientalis* females that were caught inside a house of Umsalala village. More recently, Gebresilassie, Yared, et al. [10] found *L. donovani* infection in 0.5% of 575 *P. orientalis* females collected from peri-domestic and agricultural fields in Tahtay Adiyabo district, northern Ethiopia. Kirstein et al. [24] found infection rates ranging between 3.00–23.0% in pools of *P. orientalis* flies captured in three villages in the same region of northern Ethiopia. Working in Southwest Ethiopia, Gebre-Michael & Lane [8] reported *L. donovani* natural infection rates of 0.7% (16 out of 2326) and 0.3% (3 out of 1044), in *P. martini* and *P. celiae*, respectively. In this study, we encountered an overall 1.4% infection rate of *L. donovani* in *P. orientalis*, sampled in three consecutive transmission seasons. There was a clear annual fluctuation in the infection rate, ranging between 0.0% in 2016 and 8.0% in 2017. This result may be related to annual fluctuation in density of the vector and the number of cases of the disease, which were not quantified in this study.

This is the first report of a comparison of infection rates of *L. donovani* in *P. orientalis* captured at different microhabitats of a VL endemic village. In previous studies, sand fly samples that were screened for *L. donovani* infection were either captured at one micro-habitat type or obtained from multiple sites without considering their spatial separation in calculations of the infection rates [10,15]. Although our results showed uniform infection rates in sand flies captured at different microhabitats in the study village, there was a tendency for increased proportions of infected flies in the outdoor sites. This result is not surprising, since the outdoor sites are the locations where people and their livestock and domestic animals are found during the night. With the well-known difficulty of infected flies to engorge fully in one feeding attempt [39], it is likely that infected sand flies would be hungrier and have higher foraging rates and therefore more concentration around blood-meal hosts than uninfected flies.

It is of particular interest that we found that three of the *P. orientalis* females captured at the sylvatic site were infected with *L. donovani*. Although we cannot rule out the possibility that these flies were infected by feeding on VL patients in the village, it is also possible that they received the infection from an unknown reservoir host in the sylvatic site. In previous studies, we demonstrated a zoonotic focus of *L. donovani* in an uninhabited site in Dinder National Park, located 40 km from Belo village [14]. In a subsequent study, we detected the presence of *L. donovani* in two out of 13 Egyptian mongooses (*Herpestes Ichneumon*), captured in Dinder National Park and areas adjacent to Belo village [25], indicating the potential of this animal to act as a reservoir host of the parasite. Further evidence supporting this notion was obtained by Hassan et al., who showed high attractiveness of this animal to host-seeking *P. orientalis* females [40]. It is noteworthy that the Egyptian mongoose is commonly seen in the Belo village area. Identifying this animal or other wild animals as potential reservoir hosts of VL around the villages will have important ramifications in the epidemiology and control of the disease.

It may be argued that the kDNA is not species-specific for *L. donovani* and that the infected flies were harboring other species of *Leishmania* parasites. This notion is considered unlikely in our study since *L. donovani* is the only species circulating in the area. We also ruled out this possibility by amplifying the *cpb* gene product, which matched *L. donovani.* Furthermore, we attempted to characterize the strain of *L. donovani* in the infected flies, in two specimens, by sequencing the ITS1 region of the parasite genome. The fact that we succeeded in this attempt with only two specimens was not surprising since the ITS1 region has a lower copy number, and therefore it is hard to amplify its DNA with sufficient concentrations for sequencing [41]. Nevertheless, our findings matched previously characterized *L. donovani* from Sudan. It is important to mention that there is no *L. infantum* in Sudan [42].

The overall 1.4% infection rate of *L. donovani* in *P. orientalis* captured from all sites corresponds to the 1.6% rate reported by Hassan et al. [15], who used nested PCR to screen *Leishmania* infection in 572 female *P. orientalis* captured in outdoor sites of four villages in the same area. However, comparing the results for the outdoor sites only, it appears that the infection in Belo village is higher previously reported by Hassan et al. [15].

Typing of blood meals and characterization of the blood-feeding status of the sand flies screened for *Leishmania* showed that 11 of the infected *P. orientalis* females contained fresh blood that originated from cows, humans, and donkeys. From these results, we infer that the infected females were in the active phase of searching for a host to feed on. We had previously documented that in the study area, *P. orientalis* preferentially feeds on cows, donkeys, and humans rather than other hosts [20]. Like other domestic animals, cows and donkeys are tethered inside the outdoor sites of the houses near places where children and adult people spend the night, especially in periods of the year when night temperatures are particularly high. The fact that blood found in the midguts of these infected flies was fresh blood indicates that the blood meal was taken in the same night as sand fly collection. Although it was not possible to determine whether animal blood was ingested before or after the infection, the presence of blood from cows and donkeys in the infected flies suggests a possible role of these animals in zooprophylaxis against VL. In other words, in the search for a vertebrate to feed on, infected female sand flies may bite cows and donkeys instead of people.

### Study Limitations

Due to logistic and sampling problems, the study did not include sufficient numbers of flies to allow for examination of intra-seasonal variations in infection rates in relation to climatic and other environmental variables. Also, the molecular tools employed in the study do not allow for differentiation of mature infections that are ready for transmissions. In future studies, correlations of infection rates with climatic conditions should be conducted in studies involving larger samples of *P. orientalis* sand flies that are collected on a weekly basis and examined both by microscopy and molecular techniques.

## 5. Conclusions

In conclusion, our study provides strong evidence for active transmission of VL inside villages of Gedarif state and provide an estimate of the current rate of infection of *L. donovani* in *P. orientalis* in the transmission season (March–June). The results indicate that the infected flies have a homogenous distribution in the village, with some tendency of increased infection rates in the outdoor sites. Furthermore, the findings provide evidence for the zooprophylactic role of livestock and domestic animals in reducing the risk of infection on people or indicate their possible role in transmission. While cows are non-reservoir hosts for *L. donovani*, it would be interesting to know if donkeys harbor the parasite or if they would only be acting as a “cul-de-sac”, incapable of transmitting the parasite to female sand flies that feed on them. In either case, further work is needed to expand the investigation to other villages to confirm the findings, with the aim of developing mathematical models that can help understand transmission dynamics and plan proper control programs for VL in the region.

## Figures and Tables

**Table 1:** Rates of infection of *Leishmania donovani* in *Phlebotomus orientalis* at different microhabitats and years at Belo Village, Gedarif State, Sudan.

Year/Habitat	Blood-Fed/Gravid Females		Unfed Females		All Samples		
	No. Tested	No. Positive	No. Tested	No. Positive	Total No. Tested	Total No. Positive	% Infection
**2016**							
Indoor	18	0	0	-	18	0	0.00
Outdoor	40	0	1	-	41	0	0.00
Peri-domestic	0	-	0	-	0	-	-
Sylvatic	7	0	0	-	7	0	0.00
Total	65	0	1	-	66	0	0.00
**2017**							
Indoor	30	1	0	-	30	1	3.3
Outdoor	54	8	1	-	55	8	14.5
Peri-domestic	40	0	0	-	40	0	0.0
Sylvatic	22	3	1	-	23	3	13.0
Total	146	12	2	-	148	12	8.1
**2018**							
Indoor	8	0	146	0	154	0	0.0
Outdoor	64	2	279	1	343	3	0.9
Peri-domestic	30	0	131	0	161	0	0.00
Sylvatic	11	0	195	0	206	0	0.00
Total	113	3	751	0	864	3	0.35
**All 3 Years Samples**							
Indoor	56	1	146	0	202	1	0.5
Outdoor	158	10	281	1	439	11	2.5
Peri-domestic	70	0	131	0	201	0	0.0
Sylvatic	40	3	196	0	236	3	1.7
Total	324	15	754	0	1078	15	1.4

**Table 2:** Fisher Exact Test Pairwise Comparisons of Infection rates of *Leishmania donvoani* in *Phlebotmus orientalis* sand flies captured at different sites inside and outside Belo Village, Gadarif state, Eastern Sudan.

Pairwise Comparisons	*p* Value *
Indoor vs. Outdoor	0.1159
Indoor vs. Peri-domestic	1.0000
Indoor vs. Sylvatic	0.6278
Outdoor vs. Peri-domestic	0.0207
Outdoor vs. Sylvatic	0.3991
Peri-domestic vs. Sylvatic	0.2531
Inside the village vs. Outside the Village	1.000

* Note that due to Bonferroni correction for multiple comparisons, signficant *p* values should be less than 0.0083.

## Data Availability

The molecular data that support the findings of this study are publicly available at GenBank (accession numbers provided in the manuscript). Other data are available on request from the corresponding authors.
